# The usefulness of a body shape index in assessing muscle function and strength in older adults hemodialysis patients

**DOI:** 10.3389/fendo.2025.1585193

**Published:** 2025-11-03

**Authors:** Bojan Knap, Boštjan Žvanut, Lucija Brezočnik, Mihaela Jurdana

**Affiliations:** ^1^ Department of Nephrology, University Medical Centre, Ljubljana, Slovenia; ^2^ Faculty of Health Sciences, Izola, University of Primorska, Koper, Slovenia

**Keywords:** hemodialysis, a body shape index, skeletal muscle, muscle strength and function, sarcopenia

## Abstract

**Objective:**

This study investigates the relationship between a new anthropometric measure, the Body Shape Index (ABSI), and body composition and biochemical parameters in hemodialysis patients and, for the first time, the correlation between ABSI and muscle strength and function in these patients.

**Methods:**

A cross-sectional study was conducted on a sample of 80 patients who were regularly treated in the hemodialysis unit of a single medical center of the University Hospital of Ljubljana, Slovenia. General anthropometric parameters body mass index (BMI) and ABSI=(WC/(BMI^2/3^x height½) as well as body composition data (fat mass FM, fat-free mass FFM, fat-free mass index FFMI, skeletal muscle index SMI) were determined in 25 women (aged 74.5 ± 7.5 years) and 55 men (aged 70.1 ± 6.6 years) with overweight (25 kg/m^2^ ≤ BMI < 30 kg/m^2^) and obesity (BMI ≥ 30 kg/m^2^) by bioelectrical impedance analysis (BIA). Muscle strength was determined using a hand grip strength test, while muscle performance was assessed using the sit-to-stand test.

**Results:**

ABSI was significantly negatively associated with muscle strength, functional tests and SMI only in men. Based on the median ABSI value (0.090273 m^11/6^·kg^−2/3^ in women and 0.090893 m^11/6^·kg^−2/3^ in men), women with a higher ABSI had a significantly higher glucose concentration than those with a lower ABSI. Men with a lower ABSI obtained significantly better results in the hand grip test, sit-to-stand test and waist circumference (WC).

In conclusion, our findings suggest an inverse association between ABSI and muscle strength and function in male hemodialysis patients, indicating that higher ABSI may reflect poorer physical condition in this population. Further longitudinal studies are needed to explore the clinical significance of this relationship.

## 1 Introduction

Hemodialysis is the most widely used treatment for patients with end-stage renal disease (ESRD). The prevalence of ESRD is rapidly increasing due to the aging population and increasing rates of obesity, sarcopenia, and related diseases. Given the significantly higher mortality rate associated with ESRD compared to other diseases in the general population, it is becoming a global public health concern (1, 2).

In the general population without kidney disease, several studies have shown that body mass index (BMI) and waist circumference (WC) are associated with increased mortality (3–6). “To date, relatively little research has been conducted on patients with chronic kidney disease (CKD).” When examining the relationship between BMI and WC and morbidity and mortality, these studies report contrasting conclusions: some confirm an inverse association between BMI and all-cause mortality (7–9), while others show no association between BMI and mortality (10).

However, the limitations reported in the work of the Global BMI Mortality Collaboration emphasize a heterogeneous and weak association between BMI and mortality in older populations on different continents (11). In addition, there is growing interest in finding effective and practical tests and surrogate markers that can be used in screening for early detection of populations that could benefit from targeted interventions.

Abdominal obesity (indicated by higher WC) has been identified as a potential marker associated with increased mortality in hemodialysis patients (8, 12, 13). Likewise, abdominal fat distribution (determined by WC) is also related to body size (height and weight), fat percentage and distribution. However, as WC is closely correlated with BMI, it is difficult to distinguish between the two in terms of epidemiological risk factors (14). To address this issue, the A Body Shape Index (ABSI) was introduced to overcome certain limitations of BMI and WC and provide an alternative approach to assessing body shape and associated health risks (15–17). While ABSI is based on WC, it is independent of height, weight and BMI in predicting mortality. ABSI has been associated with all-cause mortality (18) and has also been proven useful for screening metabolic syndrome (Mets) in hemodialysis patients (19). However, ABSI has not been extensively studied in relation to muscle strength and function or muscle quality. Ageing is typically associated with an increase in fat mass (FM) and a concomitant decrease in fat-free mass (FFM), leading to sarcopenia (20), an age-related clinical disorder defined by low muscle mass combined with lack of strength (21–23).

Some studies recommend measuring muscle strength in routine health assessments in clinical and epidemiological settings (24), as muscle strength declines more rapidly with age than muscle mass, even in cases when muscle mass has increased (25–27). It has recently been suggested that poor functional status is closely associated with (unfavorable) treatment outcomes (25) and increased mortality in hemodialysis patients.

Based on these considerations, our cross-sectional study investigated the relationship between anthropometric and metabolic parameters in hemodialysis patients. As, to our knowledge, the relationship between ABSI and muscle strength and function in hemodialysis patients has not yet been investigated, the study also examined the relationship between muscle strength and function and ABSI. We hypothesize that ABSI may be a useful tool for rapid screening of this condition in hemodialysis patients and investigate its potential role in routine clinical practice.

## 2 Methods

### 2.1 Study design and population

A cross-sectional study was conducted on 80 hemodialysis patients (25 women; mean age 74.5 ± 7.5 years, and 55 men; mean age 70.1 ± 6.6 years) of Caucasian origin who were regularly treated at the hemodialysis unit of the Clinical Department of Nephrology and the Department of Acute and Complex Dialysis of the University Hospital of Ljubljana, Slovenia. In our study, the dialysis prescription included 4 to 5 hours of hemodialysis in a thrice-weekly dialysis program with biocompatible hemodialysis membranes.

The following inclusion criteria were used: patients receiving hemodialysis therapy for more than 3 months, and patients receiving an adequate dialysis dose (single-pool Kt/V ≥ 1.2). Patients with clinical comorbidities (clinical signs of overt infection including COVID-19, history of cancer and renal transplantation vasculitis or liver disease, the presence of limb amputation, contraindications for BIA: patients with pacemakers, implantable cardioverter defibrillators, cardiac resynchronization therapy defibrillators, and patients hospitalized in the month prior to study inclusion) were excluded. The clinical status of each patient was evaluated by means of a routine clinical examination before each regular hemodialysis session and all measurements were performed prior to the dialysis session.

Patients were examined between April 2023 and December 2023 in the clinical nephrology department of the above-mentioned institution.

The study was approved by the Slovenian National Ethics Committee (code number KME RS 0120-179/2023/3) and was performed in accordance with the Declaration of Helsinki. Written informed consent was obtained from all participants and their rights were respected.

### 2.2 Anthropometric data

All anthropometric measurements were taken in the morning. Height (m) and weight (kg) were measured using standard methods. Body weight was measured with an accuracy of 0.1 kg (single layer clothing, without shoes). BMI was then calculated as weight/height squared (kg/m^2^). Waist circumference (WC) was measured standing, using a non-stretchable tape measure at the uppermost border of the iliac crests. A body shape index ABSI was calculated as WC/(BMI^2/3^ * height^1/2^), expressed in m^11/6^ kg^−2/3^ (15).

Patients were divided into two groups based on the median value of their individual ABSI measurements serving as the threshold. Patients whose ABSI value was lower than the median value were assigned to the “lower-ABSI” group, and patients whose ABSI value was higher than the median value were assigned to the “higher-ABSI” group.

Physical activity was estimated using information from the NPAQ-short – a brief physical activity questionnaire (28). Two questions considered suitable for monitoring the WHO recommendations for physical activity in the general population addressed the duration of physical activity in the previous week.

### 2.3 Evaluation of hand grip strength

The Jamar hand dynamometer (Patterson Medical) was used as the gold standard for the assessment of maximal voluntary grip force contraction with a maximum grip force of 90 kg at 2-kg intervals. The Jamar dynamometer was set to the second grip position to measure each patient’s grip force. To perform the test, patients held the Jamar dynamometer in the palm of their hand and pulled the metal bar towards their palm with their fingers A trained examiner explained and demonstrated the protocol to the patients. The test was performed on both hands. To ensure consistency of the procedure and minimize inter-rater variability, all measurements were performed by the same examiner. Patient performed 3 consecutive trials of grip strength per hand, with 15 s rest periods between repetitions. The highest value of the three trials was used for the analysis.

### 2.4 Sit-to-stand test

Patients were instructed to stand up as quickly as possible from a chair with seat height of 45 cm and perform sit-to-stand repetitions for 30 seconds. To ensure that the test was performed correctly, patients were instructed to have their back touch the backrest in the sitting position. The number of complete stands from the sitting position was recorded. The tests were performed without upper limb support.

### 2.5 Body composition measurements

Fat mass FM (kg), fat-free mass FFM (kg), appendicular skeletal muscle mass ASMM (kg), and phase angle PhA (°) were assessed using bioelectrical impedance analysis (BIA-101, AKERN-Srl, Firenze, Italy). Skeletal muscle mass index SMI (kg/m^2^) was calculated using the sum of skeletal muscle mass (lean mass) of both arms and legs (ASMM) divided by height squared (kg/m^2^). Patients were assessed lying in a supine position with low-impedance adhesive electrodes placed on the back of hands and feet. Fat-free mass index (FFMI) was calculated as FFM (kg) divided by the square of body height in meters (m^2^). The ratio between FM and FFM (FM/FFM) was calculated as an index of sarcopenic obesity using the following values (29): FM/FFM ratio: < 0.40 for metabolically healthy obese individuals in whom the increase in FM was small compared to FFM; FM/FFM ratios between 0.40 and 0.80 for obese patients; and FM/FFM > 0.80 for sarcopenically obese individuals in whom FM predominated over FFM.

### 2.6 Metabolic parameters

Laboratory parameters including serum glucose, total cholesterol, high density lipoprotein cholesterol (HDL), low density lipoprotein cholesterol (LDL), triglycerides (TG), creatinine, alanine aminotransferase (ALT), aspartate aminotransferase (AST), gamma-glutamyl transferase (GGT), C-reactive protein (CRP) were obtained from the hospital database. The Atherogenic Index of Plasma (AIP), a marker for the prediction of atherosclerosis and the risk of coronary heart disease, was calculated using the following equation (30): log (TG/HDL), where TG stands for triglycerides and HDL for high-density lipoproteins.

### 2.7 Statistical analysis

Univariate data analysis was performed by calculating the frequencies and percentages for nominal and ordinal variables. For numeric variables, the mean and/or median were calculated along with the standard deviation (SD). Normality of distribution was assessed using histograms, skewness, kurtosis and Kolmogorov-Smirnov and Shapiro-Wilk tests. Statistically significant differences between the two groups were determined using independent sample t-tests. In cases where there was a notable deviation from the normal distribution, the Mann-Whitney U test was used. Spearman’s rank correlation coefficient was calculated to assess the association between the variables, as most variables were not normally distributed.

*Post hoc* power analysis for multiple linear regression (five predictors, α=0.05) indicated that the overall sample size (N=80) provided adequate power to detect large effects (Cohen’s f²=0.543, 1−β=0.99). When stratified by sex, the male subgroup (n=55) also demonstrated high power (1−β=0.990), whereas the female subgroup (n=25) had limited power (1−β=0.196), which may restricts the ability to detect small to moderate effects in this group.

Correction for multiple testing was applied using the FDR method. Multiple linear regression analyses were performed to further examine the independent associations of ABSI and hand grip strength, sit-to-stand performance, physical activity, and duration of hemodialysis in sex-stratified models. For regression models, both unstandardized (B) and standardized (β) coefficients, p-values, and model fit indices (R^2^, adjusted R^2^, F) were reported. *Post-hoc* power analyses for the regression models were conducted in RStudio (version 2025.05.1+513). Effect sizes were calculated as Cohen’s f^2^ (f^2^=R^2^/(1−R^2^)) and achieved power (1−β) was reported for overall, male, and female subsamples.

Statistical analyses were performed using IBM SPSS version 29 (IBM Corp., Armonk, NY, USA) and R (R Core Team, Vienna, Austria). The significance level was set at α=0.05.

## 3 Results

### 3.1 Baseline anthropometric and biochemical characteristics of hemodialysis patients

In this cross-sectional study, a total of 80 Slovenian hemodialysis patients (25 women and 55 men, mean age 74.5 ± 7.5 and 70.1 ± 6.6 years, respectively) were examined. The general characteristics of hemodialysis patients stratified by gender are shown in **Table 1**. Female and male patients were comparable in terms of BMI, WC and serum glucose, liver enzymes AST, ALT, GGT, total, HDL and LDL cholesterol levels. Weight, height, plasma creatinine levels and FFM, FFMI, SMI and ASMM, as well as hand grip strength and PhA were significantly higher in men, while age, creatinine concentration, FM and the FM/FFM ratio were higher in women. No statistically significant differences were observed in ABSI, AIP and CRP between the groups. According to the FM/FFM > 0.80, four hemodialysis patients had sarcopenic obesity, including three women and one man. A higher percentage of women (48%) exhibited an obese phenotype, while (78%) of men were metabolically healthy obese individuals. The PhA was significantly greater in men than in women (5.2 ± 1.3° and 4.5°± 0.7°, respectively; p < 0.05) and was below the estimated reference values resulting from gender and age in both groups.

**Table 1:** Baseline characteristics of hemodialysis patients stratified by gender.

Parameters	Women (n=25)	Men (n=55)	Differences between genders (p)	Effect size	95% Confidence Interval	
					Lower	Upper
Age (years)	74.5 ± 7.5	70.1 ± 6.6	0.011^a^	-0.632	-1.113	-0.147
Weight (kg)	68.8 ± 10.9	80.2 ± 12.4	0.001^b^	-0.477	0.446	1.437
Height (cm)	160.2 ± 6.3	175.8 ± 7.4	< 0.001^b^	-0.885	1.622	2.795
BMI (kg/m2)	26.8 ± 3.7	25.9 ± 3.3	0.272^a^	-0.267	-0.740	0.208
WC (cm)	103.3 ± 13.0	104.9 ± 11.5	0.583^a^	0.133	0.341	0.605
SYS (mmHg)	150.0 ± 22.9	154.5 ± 23.9	0.436^a^	0.189	-0.286	0.662
DIA (mmHg)	75.4 ± 14.2	77.1 ± 11.6	0.663^b^	-0.061	-0.337	0.609
Sit – to- stand test (30s)	8.3 ± 6.0	10.6 ± 5.3	0.153^b^	-0.199	-0.056	0.899
Hand grip strength R (kg)	14.7 ± 4.8	28.0 ± 9.5	< 0.001^a^	1.605	1.065	2.136
Hand grip strength L (kg)	14.1 ± 5.8	25.1 ± 8.9	< 0.001^a^	1.357	0.835	1.871
Glucose (mmol/L)	5.80 ± 2.10	5.80 ± 1.80	0.779^b^	-0.039	-0.455	0.490
Creatinine (µmol/L)	695.70 ± 158.8	814.30 ± 196.80	0.010^a^	0.638	0.152	1.119
ALT (µkat/L)	0.27 ± 0.22	0.25 ± 0.12	0.864^b^	0.024	-0.661	0.286
AST (µkat/L)	0.30 0.14	0.28 ± 0.10	0.499^b^	0.094	-0.690	0.258
GGT (µkat/L	0.58 ± 0.69	0.65 ± 1.00	0.377^b^	-0.124	-0.402	0.544
Cholesterol (mmol/l)	3.98 ± 0.98	3.96 ± 1.02	0.935^a^	-0.020	0.080	1.045
HDL (mmol/L)	1.08 ± 0.36	1.06 ± 0.35	0.847^b^	0.027	-0.524	0.422
LDL (mmol/L)	2.22 ± 0.87	2.33 ± 0.91	0.614^a^	0.122	-0.351	0.595
TG (mmol/L)	1.51 ± 0.65	1.43 ± 1.02	0.090^b^	0.237	-0.561	0.385
CRP (mg/L)	8.52 ± 9.17	10.82 ± 16.63	0.613^b^	-0.066	-0.318	0.629
AIP (mg/dL)	0.12 ± 0.28	0.09 ± 0.28	0.230^b^	0.169	-0.602	0.344
FM (kg)	22.1 ± 8.2	17.4 ± 9.2	0.020^b^	0.325	-1.013	-0.053
FFM (kg)	46.2 ± 8.7	62.9 ± 7.1	< 0.001^b^	-0.879	1.591	2.757
FFMI (kg/m^2^)	18.5 ± 2.2	20.53 ± 2.0	< 0.001^a^	0.965	0.466	1.459
FM/FFM ratio	0.50 ± 0.2	0.28 ± 0.1	< 0.001^b^	0.602	-1.761	-0.737
< 0.4	10 (40.0%)	43 (78.2%)				
[0.4 - 0.8]	12 (48.0%)	11 (20.0%)				
> 0.8	3 (12.0%)	1 (1.8%)				
ABSI(m^11/6^/kg^2/3^)	0.0913 ± 0.00627	0.09057 ± 0.00553	0.913^b^	-0.015	-0.600	0.346
Physical activity (min/week)	132.0 ± 134.6	130.7 ± 9	0.555^b^	-0.082	-0.485	0.461
SMI (kg/m^2^)	8.2 ± 1.3	10.5 ± 1.5	< 0.001^b^	-0.793	1.047	2.115
ASMM (kg)	16.8 ± 2.8	23.7 ± 3.4	< 0.001^b^	-0.894	1.551	2.710
PhA (°)	4.5 ± 0.7	5.2 ± 1.3	0.020^b^	-0.327	0.080	1.045
Dialysis vintage (months)	95.1 ± 112.1	95.5 ± 78.1	0.182^b^	0.187	-0.477	0.469

data are expressed by mean values ± SD, excepted distribution of the FM/FFM ratio (< 0.4, [0.4–0.8], > 0.8) is represented as n (%), ^a^independent samples t-test; ^b^Mann Whitney U test. Effect sizes are reported as Cohen’s d for independent samples t-tests and as rank biserial correlation (r) for Mann–Whitney U tests. BMI, body mass index; WC, waist circumference; SYS, systolic blood pressure; DIA, diastolic blood pressure; ALT, alanine aminotransferase; AST, aspartate aminotransferase; Gamma GT, gamma-glutamyl transferase; HDL, high-density lipoprotein; LDL, low-density lipoprotein; TG, triglycerides; CRP, C-reactive protein; AIP, atherogenic plasma index; FM, fat mass; FFM, fat-free mass; FFMI, fat-free mass index; ABSI, a body shape index; SMI, skeletal muscle mass index; ASMM, appendicular skeletal muscle mass; PhA, phase angle.

### 3.2 Gender-specific differences in the association between ABSI, body composition, and biochemical parameters

A correlation analysis between anthropometric, biochemical and body composition parameters was performed for male (**Table 2**) and female patients (**Table 3**) separately. In both groups, BMI was positively associated with WC and all body composition parameters (FM, FFMI, FM/FFM, and only in men with ASMM, whereas ABSI was not significantly correlated with BMI in either women or men. In addition, a correlation between FM and FM/FFM was observed in all patients, while correlation between FFMI, SMI and ASMM was observed in male patients only.

**Table 2:** Relationships between anthropometric and body composition parameters for male hemodialysis patients (n=55).

Parameter	WC	ABSI	FM	FFMI	FM/FFM	SMI	ASMM	Physical activity	Sit-to-stand	Hand grip R	Hand grip L	PhA (°)	AIP	CRP	Glucose	Cholesterol
BMI (kg/m^2^)	0.775**	0.046	0.706**	0.453**	0.623**	0.090	0.423**	0.014	-0.211	-0.137	-0.184	0.063	0.255	0.190	-0.154	0.023
WC (cm)		0.562**	0.724**	0.183	0.684**	-0.194	0.270	0.066	-0.388*	-0.253	-0.346	-0.062	0.232	0.189	-0.139	0.046
ABSI (m^11/6^/kg^2/3^)			0.209	-0.256	0.275	-0.373*	-0.230	-0.007	-0.406*	-0.453**	-0.495**	-0.229	0.015	0.136	0.012	0.108
FM (kg)				-0.103	0.974**	-0.259	0.145	-0.061	-0.265	-0.181	-0.185	-0.344	0.168	0.155	-0.180	0.001
FFMI (kg/m^2^)					-0.239	0.623**	0.522**	0.086	0.313	0.240	0.164	0.646**	0.064	0.181	-0.056	-0.033
FM/FFM ratio						-0.349*	-0.021	-0.085	-0.329	-0.265	-0.239	-0.488**	0.122	0.118	-0.126	-0.004
SMI (kg/m^2^)							0.553**	0.017	0.461**	0.282	0.249	0.367*	-0.032	0.101	0.092	-0.082
ASMM (kg)								0.136	0.189	0.262	0.134	0.487**	0.111	0.044	-0.090	-0.002
Physical activity (min/week)									0.130	0.144	0.050	0.223	-0.026	-0.052	-0.150	-0.295
Sit-to-stand (30s)										0.425**	0.274	0.323	0.071	0.112	-0.091	-0.055
Hand grip R (kg)											0.783**	0.308	0.117	-0.244	0.049	0.077
Hand grip L (kg)												0.230	0.137	-0.248	0.020	0.076
PhA (°)													0.129	0.098	-0.251	0.068
AIP (mg/dL)														0.296	-0.116	0.196
CRP (mg/L)															-0.002	0.040
Glucose (mmol/L)																-0.269

*p<0.05. **p<0.01.

BMI, body mass index; WC, waist circumference; ABSI, A body shape index; FM, fat mass; FFM, fat free mass; FFMI, fat free mass index; SMI, skeletal muscle mass index; ASMM, appendicular skeletal muscle mass; PhA, phase angle; AIP, atherogenic plasma index; CRP, C- reactive protein.

**Table 3:** Relationships between anthropometric and body composition parameters for female hemodialysis patients (n=25).

Parameter	WC	ABSI	FM	FFMI	FM/FFM	SMI	ASMM	Physical activity	Sit-to-stand	Hand grip R	Hand grip L)	PhA (°)	AIP	CRP	Glucose	Cholesterol
BMI (kg/m^2^)	0.825**	-0.068	0.785**	0.495	0.602*	0.396	0.478	-0.141	-0.218	-0.236	-0.185	0.140	0.442	0.362	0.164	-0.218
WC (cm)		0.384	0.858**	0.253	0.678**	0.377	0.419	-0.386	-0.379	-0.171	-0.098	-0.225	0.353	0.600*	0.249	-0.295
ABSI (m^11/6^/kg^2/3^)			0.072	-0.262	0.006	0.085	-0.212	-0.262	-0.283	-0.014	-0.096	-0.482	0.147	0.506	0.449	-0.327
FM (kg)				0.037	0.871**	0.239	0.336	-0.342	-0.267	-0.247	-0.131	-0.139	0.195	0.302	-0.008	-0.242
FFMI (kg/m^2^)					-0.148	0.501	0.475	-0.044	-0.022	-0.057	-0.119	0.327	0.250	0.244	0.039	0.250
FM/FFM ratio						0.031	0.138	-0.297	-0.315	-0.338	-0.114	-0.276	0.146	0.144	-0.011	-0.341
SMI (kg/m^2^)							0.701**	-0.412	-0.332	-0.170	-0.209	0.003	0.099	-0.002	-0.017	0.209
ASMM (kg)								-0.433	-0.186	0.176	0.306	0.090	0.074	0.032	-0.044	0.234
Physical activity (min/week)									0.623*	0.149	-0.028	0.313	-0.240	-0.007	0.116	0.055
Sit-to-stand (30s)										0.235	0.124	0.310	-0.177	0.025	-0.072	0.027
Hand grip R (kg)											0.768**	0.285	-0.130	0.080	0.081	0.223
Hand grip L (kg)												0.020	-0.137	-0.041	0.045	0.300
PhA (°)													0.026	-0.204	-0.114	0.024
AIP (mg/dL)														0.230	0.263	-0.368
CRP (mg/L)															0.192	-0.324
Glucose (mmol/L)																-0.454

*p<0.05. **p<0.01.

BMI, body mass index; WC, waist circumference; ABSI, A body shape index; FM, fat mass; FFM, fat free mass; FFMI, fat- free mass index; SMI, skeletal muscle mass index; ASMM, appendicular skeletal muscle mass; PhA, phase angle; AIP, atherogenic plasma index; CRP, C- reactive protein.

The ABSI and body composition parameters were not significantly correlated in women, while a significant negative correlation with SMI (r=-0.373, p<0.05) and a significant positive correlation with WC (r=0.562, p<0.01) were observed in men. We also found a significant positive correlation between SMI and FFMI (r=0.623, p<0.01), suggesting that FFMI may be as a simple surrogate marker for SMI to detect low muscle mass in sarcopenia.

There was a negative association between SMI and FM/FFM ratio (r=-0,349, p<0.05), as well as a negative association between PhA and FM/FFM (r=-0.488, p<0.01), and a positive with FFMI (r=0.646, p<0.01) as well as SMI (r=0.367, p<0.05) and ASMM (r=0.487, p<0.01). In addition, the ABSI correlated negatively with the sit-to-stand test (r=-0.406, p<0.05) and the hand grip strength test (right hand: r=-0.453, p<0.01; left hand: r=-0.495, p<0.01) only in men.

Overall, a positive correlation between WC and FM, FM/FFM, was observed in both sexes. We also observed a positive correlation between WC and CRP in women (r=0.600, p<0.05). More physical activity per week seems to improve the results of the sit-to-stand test in women only.

### 3.3 A gender-specific characteristic of the lower and higher ABSI groups

To selectively analyze the relationship between ABSI and anthropometric, body composition and biochemical measurements of inflammation, muscle mass and strength independent of BMI, both male and female patients were divided into two groups using the median of individual ABSI measurements as a threshold (0.090273 m^11/6^·kg^−2/3^ in women and 0.090893 m^11/6^·kg^−2/3^ in men; see **Table 4**).

**Table 4:** Body composition and biochemical parameters in female and male patients with lower and higher ABSI.

Parameter	ABSI Women			ABSI Men		p
	Lower-ABSI	Higher-ABSI	p	Lower-ABSI	Higher-ABSI	
Age (years)	72.5 ± 7	76.3 ± 7.8	.200^b^	68.8 ± 6.0	71.5 ± 7.1	.131^a^
BMI (kg/m^2^)	26.9 ± 3.1	26.7 ± 4.3	.663^b^	25.7 ± 2.8	26.1 ± 3.8	.692^a^
WC (cm)	98.4 ± 8.2	107.8 ± 15.2	0.069^a^	100.2 ± 9.9	110.2 ± 11.0	**< 0.001^a^**
Sit to stand test (30s)	9.6 ± 5.5	7.1 ± 6.5	.238^b^	12.6 ± 4.7	8.4 ± 5.2	**.012^b^**
Hand grip strength R (kg)	15.3 ± 4.6	14.0 ± 5.1	.300^b^	31.5 ± 8.6	24.1 ± 8.9	**.004^b^**
Hand grip strength L (kg)	14.9 ± 6.5	13.4 ± 5.2	.445^b^	28.6 ± 8.1	21.1 ± 8.2	**.001^a^**
Glucose (mmol/L)	4.88 ± 0.40	6.67 ± 2.63	**.006** ^b^	5.60 ± 1.37	6.12 ± 2.13	.625^b^
CRP (mg/L)	5.75 ± 5.86	11.08 ± 11.05	.168^b^	8.31 ± 9.02	13.62 ± 22.15	.335^b^
FM (kg)	21.7 ± 8.6	22.5 ± 8.2	.811^a^	16.5 ± 8.8	18.3 ± 9.7	.561^b^
FFM (kg)	45.4 ± 9.6	47.0 ± 8.1	.765^b^	64.1 ± 7.5	61.5 ± 6.6	.172^b^
FFMI (kg/m^2^)	19.2 ± 1.8	17.9 ± 2.4	.149^a^	20.8 ± 2.0	20.2 ± 2.1	.228^a^
PhA (°)	4.8 ± 0.6	4.2 ± 0.7	.056^b^	5.4 ± 1.5	4.8 ± 0.9	.208^b^

Data are expressed in mean values ± SD; a - independent sample *t-test*, b - Mann Whitney U test. BMI, body mass index; WC, waist circumference; CRP, C-reactive protein; FM, fat mass; FFM, fat free mass; FFMI, Fat free mass index), ABSI, A body shape index. Subjects were stratified in two groups (“Lower-ABSI” and “Higher-ABSI”) according to the ABSI median value, in women (i.e., 0.090273 m^11/6^kg^2/3^) and men (i.e., 0.090893 m^11/6^kg^2/3^), as threshold. Statistically significant results are highlighted in bold.

Men from the “lower-ABSI” group obtained significantly better results in muscle function tests than men from the “higher-ABSI” group (sit-to-stand test: 12.6 ± 4.7 *vs*. 8.4 ± 5.2, U=229, p=0.012, r=0.39, 95% CI [0.02, 0.69]; right hand grip strength test: 31.5 ± 8.6 *vs*. 24.1 ± 8.9, U=205, p=0.004, r=0.46, 95% CI [0.09, 0.74] and left hand grip strength test: 28.6 ± 8.1 *vs*. 21.1 ± 8.2, t (53)=3.43, p=0.001, d=0.93, 95% CI [0.36, 1.48]), highlighting a potential association between ABSI and skeletal muscle function and strength in the extremities. WC was significantly higher in the male “higher-ABSI” group (110.2 ± 11.0 *vs*. 100.2 ± 9.9, t (53)=–3.54, p < 0.001, d=–0.96, 95% CI [–1.51, –0.39]), while the female “higher-ABSI” group exhibited significantly higher glucose concentration values (4.88 ± 0.4 *vs*. 6.67 ± 2.63, U=28.0, p=0.006). The effect size was large (r=0.64, 95% CI [0.04, 0.91]), with high *post-hoc* power (>0.90).

Multiple linear regression analysis revealed that ABSI independently predicts poorer muscle function performance (β=–366.68, p=0.004), reduced right handgrip strength (β=–783.21, p < 0.001), and reduced left handgrip strength (β=–790.99, p < 0.001) in male patients.

In females, ABSI independently predicts PhA (β=–56.18, p=0.013).

## 4 Discussion

This cross-sectional study used body composition and biochemical parameters and utilizes indexes of body composition that are easily obtained in routine clinical practice and investigated the ability of the ABSI to evaluate muscle mass and muscle strength and function as well as metabolic health in hemodialysis patients.

ABSI is a recently introduced measure of abdominal fat that may provide a better measure of central adiposity (15, 31–34). The goal of the ABSI is to predict disease risk, which cannot be captured by BMI (35). Since its introduction in by Krakauer and Krakauer (15) in 2012 to make WC statistically independent of BMI and height, ABSI has been investigated in several studies, but none to our knowledge have used it to assess muscle strength and function in hemodialysis patients. The results of our study reveal gender-specific differences in the association between ABSI, body composition, and biochemical parameters.

We found that in hemodialysis patients, BMI was positively correlated with all body composition parameters (i.e. FM, FM/FFM, WC) in both men and women, as well as with FFMI and ASMM in men. The lack of correlation between BMI and ABSI in other studies (15, 31, 32, 36) was also confirmed in our study, with no differences between genders.

ABSI could improve relevant information on abdominal obesity, body composition and mortality risk in previous studies (37, 38). In our male patients, a positive correlation was observed between ABSI and WC and a negative correlation between ABSI and SMI (an important parameter for the diagnosis of sarcopenia). Moreover, we divided our patients into two groups based on their median ABSI. Male hemodialysis patients with a higher ABSI also had a significantly higher WC. As previously described, a higher ABSI value is an indicator of higher abdominal adiposity, which leads to systemic inflammation, insulin resistance and systemic loss of skeletal muscle mass (20, 32, 36). In addition, ABSI was negatively associated with muscle strength (both hand grip strength tests, p < 0.01) and function (sit-to-stand test, p < 0.05). Men with a higher ABSI obtained significantly poorer results in the hand grip strength test. Some studies conducted on the general population show a correlation between muscle strength and sarcopenia with a higher ABSI (39, 40) and a negative correlation between hand grip strength and ABSI, suggesting that individuals with a more centralized body profile tend to be weaker than others of the same weight (22). These studies suggests that mortality is strongly associated with low hand grip strength as well as increased ABSI.

The sit-to-stand test, which is used as a measure of lower body strength and is considered a predictor of physical performance (41) and a valuable tool for the functional assessment of hemodialysis patients, was also lower in men with a higher ABSI.

This study is the first to describe muscle strength and performance and probable sarcopenia using ABSI in men. No association between ABSI and muscle mass was observed in our study. Previous studies have reported that muscle strength and physical performance, rather than muscle mass, are significantly associated with all-cause mortality in hemodialysis patients (25, 42, 43). However, in these studies, muscle mass and strength were not assessed using ABSI.

Conflicting results have also been reported. Two studies reported higher muscle mass in hemodialysis patients to be independently associated with a lower risk of all-cause mortality (44, 45). This discrepancy in the results could be explained by the difference in sample size as well as geographical differences. Strong association between sarcopenia (loss of muscle mass and strength) and mortality in hemodialysis patients has been previously reported (25). In line with more recent studies on hemodialysis patients (46, 47) and according to the criteria of the EWGSOP2 (European Working Group on Sarcopenia in Older People), probable sarcopenia, defined as reduced muscle strength (with normal muscle mass), (48) was confirmed in our male patients. This should not be interpreted as lack of importance of muscle mass over function. Given the cross-sectional study design and the absence of long-term follow-up and clinical outcomes, this finding should be interpreted as a potential risk factor rather than a definitive diagnosis. Conversely, we found no correlation between muscle function and muscle strength in women.

The direct relationships between FFMI and SMI and BMI in men may reflect the anabolic efficiency of skeletal muscle loading by body weight, as isometric contraction has been shown to be an important stimulus for skeletal muscle hypertrophy (47). Among women, there was no observed association between ABSI and biochemical or body composition parameters. However, women with higher ABSI had significantly higher glucose concentrations.

Several mechanisms could explain the negative association of abdominal fat accumulation with muscle strength and function in male patients and its positive association with glucose in female patients. Firstly, abdominal obesity is characterized by systemic inflammation, oxidative stress and insulin resistance, which may stimulate muscle protein degradation and inhibit protein synthesis (32). Secondly, physical inactivity is associated with sarcopenia and promotes the deposition of abdominal fat (49, 50). This was confirmed by higher ABSI and lower muscle strength and function in male patients and increased glucose concentration in female patients. Similar results have been observed by others in obese and sarcopenically obese subjects (32). It has been suggested that PhA may be useful to assess muscle quality and function, and it is known to be negatively affected by disease, inflammation and functional disability (51). Inflammation itself has been shown to be a strong and independent predictor of mortality in hemodialysis population (52).

In agreement with other studies (53–55), a direct correlation between PhA and muscle mass and strength, FFMI, SMI and ASMM, and a negative correlation with FM/FFM was observed in men. Further studies are needed to link PhA to muscle structure and function, as well as metabolic function. According to the FM/FFM ratio, only four hemodialysis patients were found to have sarcopenic obesity. This ratio was negatively associated with SMI, muscle function and PhA in men only, suggesting that FM/FFM may also be a determinant of the loss of muscle strength, as previously shown (54).

Finally, gender-specific differences in the observed results can be partly attributed to the influence of sex hormones (56). Estrogen has been shown to support skeletal muscle mass and function by modulating inflammatory responses, promoting muscle protein synthesis, and facilitating muscle repair and regeneration. The decline in estrogen levels is associated with increased muscle inflammation and accelerated decline in muscle mass and strength (57). Similarly, the age-related decline in serum testosterone levels correlates with a decrease in muscle mass and strength and contributes to sarcopenia in men (58).

There are several limitations to our study that should be considered when interpreting the results. Firstly, the study was conducted at a single center with a relatively small sample size, particularly among female patients, which limited statistical power and warrants cautious interpretation of the findings. Second, the cross-sectional design precludes any causal inference regarding the relationship between ABSI and muscle strength, function, or inflammation. Longitudinal studies are necessary to establish temporal relationships and causality. Third, the average age of our patients was over 70 years, which may affect the applicability of the results to younger hemodialysis patients. Fourth, while we evaluated some inflammatory markers, a more comprehensive assessment of inflammation could provide deeper insights into the observed associations, especially in female patients. Therefore, future multicenter, longitudinal studies with larger and more diverse patient populations are needed to validate our findings and further clarify the potential role of ABSI in predicting muscle mass, strength, and function in hemodialysis patients.

## 5 Conclusion

To summarize, our findings add to the growing body of research on various aspects of ABSI. Previous research has reported a positive association between ABSI and mortality, suggesting that this index may be a useful predictor of mortality risk in different populations.

Our findings suggest that ABSI may reflect reduced muscle strength and function in male hemodialysis patients, indicating its potential role in the early assessment of sarcopenia. In female patients, ABSI appears more closely related to inflammation. These results, however, are based on a single-center cross-sectional study and should be confirmed in larger, longitudinal cohorts.

## Data Availability

The raw data supporting the conclusions of this article will be made available by the authors, without undue reservation.
